# Geographical and Ethnic Heterogeneity in Genetic Dilated Cardiomyopathies

**DOI:** 10.3390/ijms27146342

**Published:** 2026-07-17

**Authors:** Matilde Di Peppo, Giovanni Biancofiore, Andrea Francesca Crudo, Gianluigi Gulino, Elena Costabile, Alessandra Margaglione, Maria Francesca D’Ambrosio, Michele Correale, Natale Daniele Brunetti, Rosa Santacroce, Maurizio Margaglione, Massimo Iacoviello

**Affiliations:** 1 School of Cardiovascular Diseases, Department of Medical and Surgical Sciences, University of Foggia, 71122 Foggia, Italy; matilde.dipeppo@unifg.it (M.D.P.); giovannibiancofiore97@gmail.com (G.B.); andrea.crudo2014@libero.it (A.F.C.); gianluigi.gulino@unifg.it (G.G.); elenacostabile@gmail.com (E.C.); natale.brunetti@unifg.it (N.D.B.); 2 Department of Clinical and Experimental Medicine, University of Foggia, 71100 Foggia, Italy; maria.dambrosio@unifg.it (M.F.D.); rosa.santacroce@unifg.it (R.S.); maurizio.margaglione@unifg.it (M.M.); 3 Cardiology Unit, Polyclinic University Hospital, Viale L. Pinto 1, 71100 Foggia, Italy; michele.correale@libero.it

**Keywords:** genetics, cardiomyopathy, geographical heterogeneity

## Abstract

Dilated cardiomyopathy (DCM) is the most common cardiomyopathy worldwide. Over the last few decades, significant progress has been made in understanding the genes responsible for DCM. A large number of variants have been detected, with heterogeneous distribution across the globe. Based on a systematic review of the available data, this review aims to investigate what we actually know about genetic, geographical and ethnic heterogeneity in DCM. What emerged is a disparity in clinical and instrumental valuations across different populations. Given the significant variability in the clinical and molecular presentation of these diseases, there is a need to develop an operational model that integrates technical and molecular diagnostics, imaging, and clinical capabilities tailored to the characteristics of different territories, while accounting for migratory flows and sex differences. By characterising specific genotypes, we could offer targeted therapies or contribute to the development of new care.

## 1. Introduction

Dilated cardiomyopathy (DCM) is defined by the presence of left ventricular dilatation and systolic dysfunction (LVEF < 50%), with or without biventricular involvement, unexplained by abnormal loading conditions, such as valvular disease, or coronary artery disease [1,2]. The prevalence has been estimated to be about 1 in 250 [3].

Among the other cardiomyopathy phenotypes, DCM is characterized by the most heterogeneous genetic architecture [4]. It is typically a monogenic Mendelian disease, most frequently with autosomal dominant transmission and incomplete penetrance. In 40% of cases, it has a monogenic aetiology [5].

Before the advent of next-generation sequencing (NGS) techniques, our knowledge was limited to a few genes tested individually in pioneering studies [3]. The incidence of these genes was around 25–30%. The discovery of NGS techniques has expanded the range of known genes and increased access to genetic testing. Moreover, an emerging concept is polygenic risk. It relates to the possible cumulative influence of numerous genetic variants, each with a small effect on an individual’s susceptibility to disease. Although individual common variants generally exert only small effects on disease risk, their aggregate contribution, quantified through polygenic risk scores (PRSs), enables the stratification of individuals across a continuum of inherited genetic risk [6].

From a clinical point of view, growing evidence shows that identifying the genotype of a disease can lead to more accurate outcome evaluation. Stroeks et al. [7] have demonstrated in a multicentre observational study of 534 patients with DCM that genotype-based risk stratification was more accurate then a phenotype-first approach.

Thanks to the latest disclosures, it has emerged that there is territorial heterogeneity in both variants and ambient interactions. Even sex differences contribute to increasing heterogeneity. In this context, it is important to differentiate between “genetic DCM” and “familial DCM,” as these terms are sometimes used interchangeably. From a clinical genetics perspective, they are distinct. A patient may have a genetic cause for their condition due to a de novo pathogenic variant, even without any family history. On the other hand, familial DCM refers to cases with multigenerational inheritance, but it may not always result in an identifiable genetic variant based on current testing panels.

This review aims to investigate the current evidence on genetic, geographical, and ethnic heterogeneity in DCM. We conducted a systematic literature search of PubMed, Embase, and the Cochrane Library for studies published between January 2000 and 2026. The search strategy included the terms “dilated cardiomyopathy,” “genetics,” and “European,” used individually or in combination. We included clinical studies, clinical trials, observational studies, systematic reviews, and meta-analyses. A total of 618 records were identified; 119 studies met the eligibility criteria and were included in the review.

## 2. The Genes Involved in DCM and Their Clinical Relevance

Most of the genes implicated in DCM encode proteins of the sarcomere, Z-discs and cytoskeleton (Table 1). Several mutated genes have been identified in DCM. When at least two closely related (first-degree or second-degree) family members meet diagnostic criteria for idiopathic DCM, the presence of familial DCM is defined [3]. The presence of a genetically determined DCM may not necessarily be related to the presence of other family members, because it could be due to a de novo pathogenic variant without any family history. Moreover, although the genetic bases of familial DCM have been established, many cases present as sporadic.

The genes most commonly known include TTN (Titin—15–20%) [8,9,10,11], LMNA (Lamin A/C—6%) [12], MYH7 (beta-myosin heavy chain—4%) [13,14], TNNT2 (Troponine T—3%) [13,15], BAG3 (Bcl2-associated athanogene 3—around 2–3%) [16,17], RBM20 (RNA binding protein 20—around 2%) [18], FLNC (Filamin C—2–4%) [19,20], SCN5A (Sodium channel protein type 5 subunit α—2%) [21,22], PLN (Cardiac phospholamban—<1%) [23,24,25], TNNC1 (Troponin C—<1%) [26,27], TNNI3 (Troponin I—<0.1%) [28,29], and TPM1 (Tropomyosin-α1 chain—<1%) [30,31]. Penetrance is commonly incomplete, which is why in some individuals who are variant carriers, cardiomyopathy does not manifest. Mutations in genes encoding cardiac troponins, such as TNNT2, TNNC1 and TNNI3, can be directly associated with a disorder in force generation [15,32]. MYH7 mutations can lead to disrupted actin–myosin binding and cross-bridge function. TTN mutations alter viscoelastic properties [33]. The domain location of truncation helps explain phenotypic consequences in DCM. A-band TTNtv produced a more overt “DCM-like” functional phenotype, with impaired sarcomere performance and a blunted adaptive response to mechanical and β-adrenergic stress, supporting sarcomere insufficiency as a disease mechanism. By contrast, I-band TTNtv was comparatively well tolerated, consistent with the greater extent of alternative splicing in the I-band, which can reduce the effective impact of some truncations at the transcript level [34]. BAG3 is positioned as a central hub of the chaperone-assisted selective autophagy complex, coupling chaperone engagement and substrate ubiquitination to the autophagic clearance of damaged sarcomeric proteins; the co-occurring P63A and P380S variants are associated with markers of dysregulated autophagy, increased protein ubiquitination, and loss of key myofilament-localised co-chaperones/adaptor proteins, culminating in reduced maximal calcium-activated force and sarcomere dysfunction [35]. These alterations can lead to left ventricular dysfunction. Some mutations may involve conduction disorders: this is the case of the LMNA mutations, whose presence increases the risk of malignant ventricular arrhythmias and of sudden cardiac death [36]. LMNA mutations lead to a defect in mechanosignalling [37,38]. LMNA cardiomyopathy typically presents initially with electrical issues, such as atrioventricular conduction abnormalities and atrial arrhythmias. Left ventricular systolic dysfunction and dilation may develop later. It is crucial to note that ventricular arrhythmias and sudden cardiac death (SCD) can occur before the clear onset of DCM, indicating that ejection fraction alone is not sufficient for risk stratification [39]. Most of the available evidence regarding this condition comes from cohorts of European ancestry and Asian populations. Although mostly associated with right ventricular cardiomyopathy, the variants in desmosomal proteins, including desmocollin 2 (encoded by DSC2), desmin (encoded by DES), desmoplakin (encoded by DSP), desmoglein 2 (encoded by DSG2), and plakophilin 2 (encoded by PKP2), are also implicated in the DCM phenotype [40]. The PLN gene encodes phospholamban, a 52 amino acid residue transmembrane protein that inhibits sarcoplasmic reticulum Ca2+-ATPase (SERCA) when it is unphosphorylated. RBM20, an RNA-binding protein, regulates cardiac splicing, including the splicing of TTN [25,41]. Hence, RBM20 mutations may be similar in their effects to those arising from TTN-truncating variants. It has been shown that a mutation in RBM20 leads to DCM through mis-splicing of TTN. This results in a transition from the stiff N2B titin isoform to the N2BA isoform, which is more compliant and ultimately leads to impaired myocardial mechanics, including a reduced Frank–Starling response [42].

**Table 1:** Major DCM genes, prevalence, inheritance patterns and associated phenotypes.

Major Genes	Inheritance Pattern	Estimated Prevalence Among DCM Cases	Major Associated Phenotypes [1]
TTN	AD	15–20% [8,9,10,11]	DCM, HCM, ARVC, NDLVC
LMNA	AD	6% [12]	DCM, ARVC, NDLVC
MYH7	AD	4% [13,14]	DCM, HCM, ARVC, NDLVC, RCM
TNNT2	AD	3% [13,15]	DCM, HCM, ARVC, NDLVC, RCM
BAG3	AD	3% [16,17]	DCM, HCM, RCM
FLNC	AD	2–4% [19,20]	DCM, HCM, ARVC, NDLVC, RCM
RBM20	AD	2% [18]	DCM, NDLVC
SCN5A	AD	2% [21,22]	DCM, ARVC, NDLVC
PLN	AD	<1% [23,24,25]	DCM, HCM, ARVC, NDLVC
TNNC1	AD	<1% [26,27]	DCM, HCM, ARVC
TPM1	AD	<1% [30,31]	DCM, HCM, ARVC, NDLVC, RCM
TNNI3	AD	<0.1% [28,29]	DCM, HCM, ARVC, RCM

AD, autosomal dominant; ARVC, arrhythmogenic right ventricular cardiomyopathy; DCM, dilated cardiomyopathy; HCM, hypertrophic cardiomyopathy; NDLVC: non dilated left ventricular cardiomyopathy; RCM, restrictive cardiomyopathy; TTN, titin; LMNA, lamin A/C; MYH7, myosin heavy chain 7; TNNT2, cardiac troponin T; BAG3, BCL2-associated athanogene 3; FLNC, filamin C; RBM20, RNA-binding motif protein 20; SCN5A, sodium voltage-gated channel alpha subunit 5; PLN, phospholamban; TNNC1, cardiac troponin C; TPM1, tropomyosin alpha-1 chain; TNNI3, cardiac troponin I.

## 3. Factors Influencing Genetic Epidemiology

### 3.1. Methods of Detection

Next-generation sequencing methods, such as whole-exome or whole-genome sequencing, are emerging as moderately inexpensive approaches for diagnosis. Landry and Rehm [43] tried to evaluate whether DCM genetic detection was different between white people, Asian people, and underrepresented minorities (individuals of black, Hispanic, Native American, Alaska Native, or Pacific Islander descent). What emerged is that the positive detection rate (pathogenetic or likely pathogenic variants) was higher in white individuals in comparison with underrepresented minorities, thus suggesting a greater clinical utility of DCM genetic testing in white persons in comparison with people of other racial/ethnic groups. However, other studies are needed to clarify this disparity and understand the gaps in utility, which may stem from a lack of clinical testing and research in underrepresented minority populations. The hope is to improve DCM-related genetic testing among non-white groups to ensure the same availability of care to all populations.

### 3.2. Gender

Gender differences still need to be clarified. In general, DCM is less represented in females, probably due to a low penetrance [44,45]. In males with TTN and LMNA variants, a greater risk of malignant arrhythmias has been observed [5,46]. For this reason, in LMNA carriers, male gender is considered to indicate the implantation of a cardioverter–defibrillator (ICD) [47]. BAG3 variants are associated with earlier onset and worse prognosis in males than females [48]. Limited evidence suggests that the FLNC variant is associated with fewer major cardiovascular events in women [20]. However, there is a need for more studies to clarify gender differences (for example, in a UK cohort, females with the FLNC variant had higher rates of events related to heart failure (HF) and of cardiovascular death [49]). Sex hormones influence cellular processes by regulating gene expression and post-translational protein modifications [50]. Androgens, oestrogens, and progesterone are particularly important in cardiovascular physiology and pathology. In the heart, oestrogen and oestrogen receptor signalling control the expression of various genes that promote reparative remodelling, as opposed to fibrosis, which leads to ventricular remodelling and failure.

### 3.3. Race and Migration

As already mentioned above, the majority of genetic DCM is inherited in an autosomal dominant pattern with variable expressivity and penetrance. De novo mutations also contribute to genetic DCM. A novel mutation is considered pathogenic when it introduces a protein-altering change in a DCM gene.

Some variants are more prevalent in certain territories. This is thought to be due to the founder effect, which occurs when a small group of individuals breaks away from a larger population to colonise a new one. This “fraction” carries only a portion of the original genome, thereby increasing the frequency of some rare mutations. This is the case for the p.Arg14del mutation in PLN, which is associated with DCM and appears to be a founder mutation in the Netherlands and Germany. Individuals from the Netherlands with the p.Arg14del founder mutation have a worse phenotype [51], whereas other studies report a milder phenotype [23,52]. The fact that the same primary mutation is associated with a range of phenotypes supports that other factors, including a different genetic background, may modify the outcome of DCM. The PLN p.Arg14del variant originated in the eastern Friesland region of the Netherlands approximately 600–800 years ago and subsequently spread, suggesting migratory flows both within the same region and internationally [53]. It would be interesting to understand how ancestral migratory flows have modified the genetic architecture. In recent years, emerging evidence suggests that the genetics of DCM is not simply the same disease across populations, but rather reflects distinct molecular architectures based on genomic ancestry [54]. A previous study highlighted how some variants considered pathogenic for hypertrophic cardiomyopathy (HCM)/DCM were relatively common polymorphisms in African Americans [55]. This underscores the need to sequence the genomes of diverse populations to avoid misclassifying benign variants as pathogenic.

### 3.4. Geographic Regions

Geographic and ethnic differences imply variations in the prevalence of DCM. Different estimations in DCM prevalence have been reported across America, Europe, Asia, Africa, and Oceania over the years [56,57], but it is likely that it is underestimated. An emerging idea is that some variants may contribute to DCM almost exclusively in certain populations. For example, CD36 loss-of-function (LOS) variants associated with DCM in individuals of African ancestry are probably linked to evolutionary selection/malaria [58]. However, the data about this topic are lacking and more studies are needed.

The adoption of current American College of Medical Genetics and Genomics and the Association for Molecular Pathology (ACMG/AMP) standards and ClinGen curation have made the reported “geographic prevalence” of DCM mutations less dependent on the composition of genetic panels and historical overcalling, resulting in a more accurate reflection of the true pathogenic and likely pathogenic (P/LP) yield [59,60]. Registries that previously used broader gene panels often reported a higher mutation burden, primarily because these expanded panels increase the likelihood of identifying variants that do not meet stringent evidence thresholds. It is also advisable to use ancestry-matched allele frequencies whenever possible. Variants that are relatively common in a specific population or region are unlikely to be truly pathogenic for a rare disorder like DCM and may be misclassified otherwise [55]. Different populations feature distinct variant spectra and genetic contexts, which include factors such as founder effects, consanguinity, and the availability of ancestry-matched controls. These factors can influence the apparent relationship between genes and phenotypes—as well as the resulting estimates of penetrance and expressivity—for genes like MYH7, TNNT2, and others related to cardiomyopathy. Geographic and ethnic factors can modify the apparent phenotypic “switch” (such as between HCM, DCM, restrictive cardiomyopathy, RCM, and arrhythmogenic cardiomyopathy, ACM) among carriers of MYH7, TNNT2, and DES variants. This is primarily influenced by differences in allele frequency, founder effects [61], consanguinity, the presence of additional rare variants (oligogenic background) [62], ancestry-dependent polygenic modifiers [63], and potential misclassification or misinterpretation of variants when ancestry-matched control data are not available [55].

### 3.5. Gene–Enviroment Interaction

DCM-promoting environmental factors are alcohol, chemotherapy, drug misuse, myocarditis, pregnancy and others.

The two-hit hypothesis [64,65] posits a model in which genetic predisposition to DCM can mediate different responses depending on environmental factors, and, reciprocally, environmental exposure might influence gene expression. This interaction has been especially observed in acquired DCM with TTNtv, like in patients with cancer therapy-induced cardiomyopathy or peripartum cardiomyopathy [66,67].

Viral infection or other conditions resulting in myocardial inflammation can be responsible for presentation of DCM. In genotype-positive patients undergoing endomyocardial biopsy, it is possible to detect the presence of subclinical inflammatory infiltrates and/or viral genomes, suggesting that they may contribute to disease progression [68].

A higher incidence of adverse cardiovascular events, in particular recurrent myocarditis and ventricular arrhythmias, has been observed in patients with myocarditis and P/LP variants in desmosomal genes (mainly DSP) when compared to unaffected patients [69,70]. Specific P/LP genetic variants likely increase the susceptibility to the onset of concomitant myocarditis in patients with ARVC, as the first clinical presentation of the disease [71,72].

A cohort of 128 probands with familial DCM has recently been investigated for DCM-promoting environmental factors. In genotype-positive/phenotype-positive individuals, a significantly higher proportion of individuals with ≥1 DCM-promoting factor was observed in comparison with genotype-positive/phenotype-negative and genotype-negative/phenotype-negative individuals [73].

More studies will be needed to understand which environmental factors influence the variants and their mechanisms of action, considering that individuals who carry a variant may not develop the disease.

### 3.6. Available Registries

**Figure 1:**
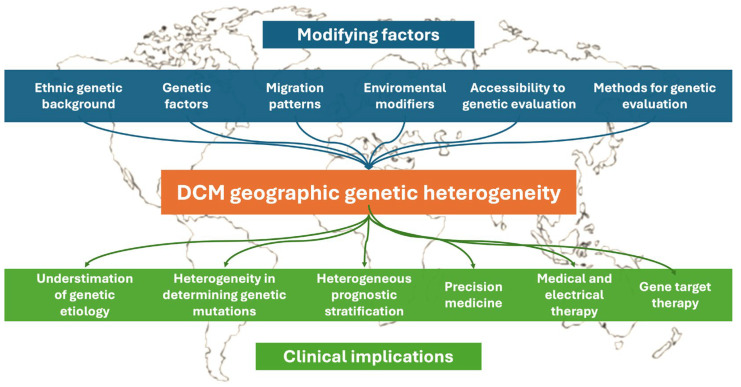
Factors determining geographic heterogeneity of patients affected by dilated cardiomyopathy and their consequences. DCM: dilated cardiomyopathy.

Finally, when geographical and ethnic heterogeneities are considered, it is worth noting that they largely depend on the available registry data, which are distributed heterogeneously across the world. Table 2 summarises the main available registries, while Figure 1 shows all of the above-mentioned influencing factors.

## 4. Genetic Epidemiology

### 4.1. Europe

There are large European and national registers that collect information. Among the main ones, we could cite the ESC EORP Cardiomyopathy registry [75] and the Heart Muscle Registry of Trieste [74]. The concentration of variants varies between the territories. For example, in the previously cited study [7], they observed differences in the genetic spectrum across centres, with TTN variants being more prevalent. Variants of TTN and LMNA were found in the cohort from Denver and Maastricht, while variants of FLNC and MYH7 were distributed among patients from Madrid and Trieste. The prevalence of PLN is mainly due to a founder mutation (p.Arg14del) that is particularly common in the northern Netherlands [81]. Additionally, a significant group of BAG3 patients was identified within the Madrid cohort, while individuals with a specific variant of the TNNT2 gene were more frequently found in the Trieste cohort.

In a study that has investigated the genetic architecture in Poland [76] in a population of 280 DCM patients, it has emerged that variants of TTN (18%) and LMNA (8%) were more frequent, while variants less represented were in genes like FLNC, SCN5A, BAG3, DSP, MYH7, and DMD.

### 4.2. North America

Many registries investigate DCM in North America. Among the most important, is the DCM Precision Medicine Study from the DCM Consortium. This registry aimed to test whether DCM has a substantial genetic basis and to identify probands and relatives to conduct clinical screening. The study enrolled 1265 DCM patients and nearly 2000 of their relatives [78]; 42% of patients were of African ancestry, and 56% were of European ancestry. The DCM Precision Medicine Study is a cross-sectional study of families at 25 US clinical sites; the cohort is multiracial, multiethnic and geographically diverse. North American DCM registries have identified TTN-truncating variants as the predominant genetic substrate of dilated cardiomyopathy, followed by LMNA, FLNC, DSP, RBM20, and PLN variants [82,83]. The prevalence of familial DCM among DCM probands was evaluated by Huggins et al. [78]. The age-specific cumulative risk of DCM was estimated in first-degree relatives across race and ethnicity groups [78]. In this study, the familial DCM prevalence was higher in Black than in white probands and was not significantly different in probands of Hispanic compared with non-Hispanic ethnicity. The estimated cumulative risk of being diagnosed with DCM increases with age, and this risk is higher among first-degree relatives of Black probands. This finding aligns with existing research on cardiovascular risk in the Black population. For instance, incident heart failure, particularly when accompanied by systolic dysfunction, is more commonly observed in Black individuals before the age of fifty [84,85]. Additionally, Black individuals with hypertension who participated in the HyperGEN study were more likely to experience a significant reduction in left ventricular ejection fraction [86]. Currently, there is no clear explanation for this phenomenon. The central hypothesis of the DCM Precision Medicine Study posits that most cases of DCM, whether familial or nonfamilial, have a genetic basis. Consequently, Black populations might possess an additional genetic risk. However, it is important to note that environmental factors, in addition to genetics and biology, can also impact the presentation and progression of the disease. The increased risk of heart failure in Black individuals has been attributed to a higher incidence of hypertension, diabetes, and other negative social determinants of health. The challenge of evaluating DCM in the North American population stems from the significant heterogeneity in ancestry resulting from migratory flows.

### 4.3. South America

There are no large South American genomic registries for DCM comparable to those in Europe or North America. A recent prospective observational study on paediatric cardiomyopathies [87] was conducted in Brazil due to the lack of data. The objective was to evaluate the clinical and genetic characteristics of paediatric DCM patients and identify mortality predictors in a metropolitan region of Brazil. In another recent study conducted in Colombia, the authors investigated primary cardiomyopathy in paediatrics: in a cohort of 76 patients, DCM was the most common subtype, and the most commonly identified P/LP variants across different morphological subtypes were MYH7, FLNC, TTN, and MYBPC3 [88]. TTN mutations and their variant interpretations have been described in patients with heart disease in Ecuador, including DCM [89].

It should be remembered that overlap with acquired cardiomyopathies, such as Chagas myocarditis, can complicate genotype–phenotype interpretation. In a multicentre study published in 2005 (The ICD-Labor Study) [90], which described the Latin American experience in the secondary prevention of SCD, the aetiology of cardiac disease was predominantly attributed to Chagas disease rather than to idiopathic dilated cardiomyopathy.

### 4.4. Africa

In accordance with the findings of the previous study [78], which reported a higher DCM prevalence among Black populations in North America, another recent study [54] investigated differences in the genetic architecture between African and European ancestries. They analysed data among 1198 patients from the DCM Precision Study.

Recent findings indicate that patients of African ancestry tend to have fewer clinically actionable genetic variants. However, it is well-documented that DCM is more prevalent among Black patients, with an earlier onset and greater morbidity and mortality rates [89,91]. Notably, TTN variants are more commonly observed in these patients. This prevalence can be attributed partly to a lower frequency of LOF variants in TTN, a variant that is more frequently represented, as well as to a lack of clinical genetic case data necessary for variant interpretation. Additionally, the absence of specific ancestral data likely hindered the classification of certain genetic variants, preventing some from being upgraded from variants of uncertain significance to P/LP. Notably, data on DCM cases in individuals of African descent are virtually non-existent in the scientific literature and minimally represented in publicly accessible clinical genetics databases [92]. This situation highlights that current understanding of the genetic architecture of DCM has predominantly relied on data from European-ancestry cohorts, which have been inappropriately applied to patients of African ancestry and their families without proper validation.

To address this gap, there is a pressing need for large-scale epidemiological studies focusing on the incidence, prevalence, and determinants of cardiomyopathy in Africa. Such research would greatly inform strategies for treating and preventing heart muscle disease on the continent. For example, a common mitochondrial DNA polymorphism (T16189C) has been identified as a genetic risk factor for DCM in a South African cohort [93]. The recent IMHOTEP registry [79], which focuses on African cardiomyopathy and myocarditis, aims to systematically collect data on individuals diagnosed with cardiomyopathy across Africa. Patients have been recruited from three centres in South Africa and one in Mozambique. Within this registry, DCM has emerged as the most common type of cardiomyopathy, with a younger age of onset that occurs more frequently in women and individuals of African ancestry compared to their European and North American counterparts. The observed gender discrepancies in DCM are largely driven by a significantly higher proportion of peripartum cardiomyopathy (PPCM) cases in Black African and mixed-race groups. Ultimately, it is clear that the absence of large registries akin to those found in Western populations results in an underestimation of the incidence and prevalence of DCM. Additionally, it is crucial to consider the potential for greater gene–environment interactions due to prevalent infections in Africa, such as Toxoplasma gondii and HIV, which may play a role in the pathogenesis of DCM.

### 4.5. Asia

A recent study conducted in the Chinese population involved whole exome sequencing (WES) genetic screening for 208 unrelated patients with DCM who were undergoing transplantation [94]. The combined analysis of truncating and non-truncating variants revealed that protein-altering variants were present in the TTN, FLNC, and LMNA genes among DCM patients. Specifically, the frequencies of truncating variants in the TTN and FLNC genes within this cohort were 18.8% and 8.7%, respectively. In another study carried out in Wuhan, researchers assessed the clinical significance of TTN gene variants [95]. They discovered that the phenotype of DCM resulting from truncating variants in the TTN gene is not distinct from that caused by other DCM-associated genes. A separate study from Japan investigated a cohort of 123 paediatric patients to examine the relationship between genotypes and clinical outcomes related to DCM, including the phenomenon of left ventricular reverse remodelling [96]. The most commonly identified gene in this study was MYH7 (14.0%), followed by RYR2 (12.0%) and TPM1 (8.0%). Additionally, a Korean study involving 72 patients with cardiomyopathies found variants in the TTN, LMNA, and MYH7 genes to be associated with DCM [97]. Furthermore, a specific variant in the MYBPC3 gene was identified in India and South Asia [98].

Table 3 summarises the available CMD genetic data across the continents.

### 4.6. Oceania

Some studies investigated dilated cardiomyopathy in childhood; the data are from the National Australian Childhood Cardiomyopathy Study, a register from 1987 to 1996 which collected 314 new cases of primary cardiomyopathy. DCM represented 58.6% [90]. Four centres in Melbourne [80] performed WES on 83 patients with idiopathic or familial DCM and classified variants using American College of Medical Genetics and Genomics–based criteria. They found 12% of P/LP variants (TTN, TNNT2, DSP, RYR2, LMNA, BAG3, and ACTC1). There are also a few studies from the Cardiac Inherited Diseases Registry New Zealand (CIDRNZ), especially evaluating sudden cardiac death in cardiomyopathies and channelopathies.

Another retrospective study evaluated Maori and non-Maori patients who underwent heart transplantation in New Zealand. Although disparities in health care access between Maori and non-Maori have been described [100], the proportion of Maori who have undergone heart transplantation in New Zealand compares favourably with their proportion of the New Zealand population [101].

## 5. Genetic Variants, Geography and Therapeutic Targets

Emerging evidence shows that identifying the genotype of a cardiomyopathy is more accurate at defining the pathology than the phenotype-first approach. This allows for risk stratification and the identification of targeted therapies (Table 4). Thanks to NGS techniques, we have made progress in diagnostics and increased accessibility to genetic testing. This is true, but it is not applicable in all cases. Current understanding of the genetics of DCM has primarily been based on populations of European ancestry. This focus may limit our ability to interpret disease-associated variants in underrepresented groups. Emerging evidence indicates that the genetic architecture of DCM varies significantly among different ancestries, with notable differences in the prevalence of TTN truncating variants and the burden of rare missense variants, as well as the availability of evidence regarding pathogenicity. Additionally, a review of the data reveals disparities in clinical and instrumental evaluations across different populations. This is particularly evident in African ethnicity, with a possible limitation in the access to care [102,103,104], including genetic evaluation and testing [43,105]. This bias creates a partial knowledge base that does not reflect global genetic diversity. This imbalance is not limited to the initial research phase but propagates through the entire translational pipeline. From the discovery of genetic variants to their clinical validation, these accumulated disparities risk making medical innovations less effective or even misleading for non-European populations [104]. Socioeconomic determinants such as income, education, and employment often affect a person’s ability to engage in preventive care and their opportunity to receive treatment. The social context in which an individual lives also strongly influences access to health care, both through the resources available in the local area and through cultural factors. Another important factor is the language barrier for minorities living in different countries, which can substantially affect risk understanding and, ultimately, adherence to therapy. Moreover, diagnostic and predictive tools currently available have been validated. They are more accurate in populations of European ancestry than in underrepresented groups, thereby increasing the risk of incorrect diagnoses in these patients [102,103]. Therefore, there is a need to develop an operational model that combines technical and molecular diagnostics, imaging, and clinical capabilities tailored to the characteristics of different territories, accounting for migratory flows and sex differences. This could help improve the diagnosis of cardiomyopathies and provide targeted therapies. Nowadays, there are risk stratification scores, some of which are still being validated, that can lead to a more accurate estimate of associated adverse event variants [46,81,106,107].

Arrhythmia management in patients with genetic DCM follows general recommendations for preventing SCD and ICD implantation based on reduced LVEF (<35%). However, specific variants correlate with increased risk of sudden cardiac death, and patients who are carriers of these mutations may benefit from ICD implantation. Through understanding molecular mechanisms and altered pathways, novel therapies have been discovered. Myosin modulators have shown promising results in treating HCM. The EXPLORER-HCM trial demonstrated that mavacamten reduces maximal actin-activated myosin ATPase activity [108]. Mavacamten is currently approved by both the FDA and the European Medicines Agency for patients with obstructive HCM. Another myosin modulator, omecamtiv mecarbil, exhibited modest benefits for patients with heart failure (HF) with reduced ejection fraction, as shown in the GALACTIC-HF trial [109]. However, it did not receive FDA approval due to an unfavourable risk–benefit ratio. Additional myosin modulators, such as danicamtiv, are currently under investigation.

Concerning LMNA variants, phase I and II trials have demonstrated that inhibiting p38 mitogen-activated protein kinase with ARRY-371797 may enhance the signs and symptoms of cardiac dysfunction [110,111]. In vitro studies using human induced pluripotent stem cell-derived cardiomyocytes (iPSC-CMs) with FLNC-truncating variants (FLNCtv) revealed that the delocalization of connexin 43 from the cell membrane leads to its nuclear translocation, which activates the platelet-derived growth factor receptor α (PDGFRA) pathway, resulting in arrhythmias [113]. These findings were corroborated by observing explanted hearts from patients with FLNCtv. Treatment with the PDGFRA inhibitor crenolanib improved both the contractile function and the arrhythmic profile of FLNCtv iPSC-CMs [112], suggesting that pharmacological inhibition of the PDGFRA pathway could be a viable therapeutic strategy for DCM caused by FLNCtv. Recent advancements have also been made in therapies aimed at precisely correcting genetic defects. This includes new, highly effective vectors; innovative capsids of adeno-associated virus (AAV) vectors with lower immunogenicity; alternative delivery systems, such as extracellular vesicles (exosomes) derived from autologous iPSC-CMs [117]; and the use of modified RNA and circular RNA as therapeutic tools [118]. A preclinical study for LMNA cardiomyopathy utilizing short hairpin RNA (PHL-001) has successfully promoted gene silencing [112]. Moreover, trials on gene replacement therapy for other DCM-associated genes, including BAG3 (AVB-401 in phase 0) and PKP2 (TN-401 in phases 0 and Ib), are currently underway [112]. Promising investigations in mice have also shown the feasibility of preventing disease onset in cardiomyopathies associated with MYH7 [114,115] or RBM20 [116] variants. While precision genome editing appears highly promising, it is crucial to conduct human trials with caution, given the safety concerns related to potential off-target effects and germline genome editing [119].

## 6. Conclusions

DCM is the most common cardiomyopathy worldwide. Over the last few decades, significant progress has been made in understanding the genes responsible for DCM, with heterogeneous distribution across the globe. There is also a disparity between clinical and instrumental evaluations across populations, primarily due to limited access to care. Operational models that combine technical and molecular diagnostics, imaging, and clinical capabilities, tailored to the characteristics of different territories, are still lacking. The goal for the future is to define operational models that help clinicians individualise the best treatment for patients.

## Figures and Tables

**Table 2:** The main available registries evaluating genetics in cardiomyopathy.

Name	Type (Registry/Cohort/Consortium)	Main Phenotype/Focus	Geography/Network	Key Features/Notes
Trieste Heart Muscle Disease Registry [74]	Single-centre longitudinal registry	Idiopathic and familial DCM (adult and paediatric), natural history, conduction disturbances, beta-blocker therapy outcomes	Trieste, Italy	Historic, deeply phenotyped DCM registry with >30-year follow-up, widely cited for natural history and prognosis in idiopathic and familial DCM
ESC EURObservational Research Programme Cardiomyopathy/Myocarditis Registry (EORP) [75]	Multicentre international registry	Adult cardiomyopathies (HCM, DCM, RCM, ARVC) and myocarditis; also includes genetic testing and management data	ESC network (>20 European countries)	Largest “real-world” European registry for cardiomyopathies, capturing contemporary management, phenotypes, and outcomes across subtypes
Polish Genetic DCM Registry (Central European DCM cohort) [76]	National DCM registry with genetic testing	Adult DCM with comprehensive gene panels (TTN, LMNA, DSP, MYH7, SCN5A, FLNC, BAG3, etc.), clinical characteristics and prognosis by genotype	Poland (multicentre national)	Provides genetic architecture and gene-specific prognosis in a Central European DCM population; useful for genotype-specific risk comparisons
DCM Precision Medicine Study [54,77,78]	US multisite family-based registry/study	Idiopathic/genetic DCM (probands and first-degree relatives), rare variant genetics, imaging, environmental modifiers	US advanced HF/Heart Failure Society programs (multiple centres)	Deep genotyped DCM cohort with standardized phenotyping; multiple analyses on variant interpretation, advanced DCM, CMR, PPCM vs. DCM, and alcohol/viral triggers
IMHOTEP—African Cardiomyopathy and Myocarditis Registry Program [79]	Pan-African multicentre registry	Cardiomyopathies (DCM, HCM, ACM, RCM, LVNC) and myocarditis in African children and adults; aetiology, genetics, management, outcomes	Sub-Saharan Africa (South Africa, Mozambique and other African centres)	Large African cardiomyopathy/myocarditis registry; DCM is the most common phenotype (≈70%); captures idiopathic, familial and secondary causes, with strong CMR and PPCM sub-analyses
National Australian Childhood Cardiomyopathy Study (NACCS) [80]	National population-based cohort	Childhood cardiomyopathies (predominantly DCM); epidemiology, aetiology and outcomes	Australia (national paediatric network)	Nationwide population-based childhood cardiomyopathy cohort. DCM was the predominant phenotype (~59%) and the study established incidence, clinical characteristics and long-term outcomes of paediatric DCM

ACM: arrhythmogenic cardiomyopathy; LVNC, left ventricular non-compaction; PPCM: peripartum cardiomyopapht; for the other abbreviations see Table 1.

**Table 3:** Geographic distribution of key DCM-associated P/LP variants. The percentage has been calculated by relating the number of P/LP variables to the number of patients. The interpretation of these data should be cautious and considered only exploratory, as the results were self-reported by investigators who used various sequencing methods [57].

Gene	Europe [74,75,76]	North America [77]	South America	Asia [94,96]	Africa	Oceania [99]
TTN	5.36%; 18%; 18.2%	8.2%	-	19.7%; /	-	4.8%
LMNA	9.78%; 5%; 7.9%	2%	-	3.8%; /	-	1.2%
MYH7	16.67%; 12% *; 3.6%	-	-	/; 5.7%	-	-
TNNT2	5.48%; 12% *; 1.4%	1%	-	-	-	1.2%
BAG3	1.79%; 11% ^; 2.5%	1%	-	1.9%; /	-	1.2%
FLNC	2.56%; 11% ^; 2.5%	2%	-	7.7%; /	-	-
RBM20	/; 6% °; 0.4%	1%	-	-	-	-
SCN5A	8.69%; 6% °; 2.9%	-	-	-	-	-
PLN	1.52%; 6% °; 0.7%	-	-	-	-	-
TNNC1	0%; 12% *; 0.4%	-	-	-	-	-
TPM1	6.34%; 12% *; 0.4%	-	-	/; 8%	-	-
TNNI3	0%; 12% *; 0.4%	-	-	-	-	-

* Cumulative percentage of sarcomeric genes. ^ Cumulative percentage of Z disk genes. ° Cumulative percentage of others genes. P/LP pathogenic/likely pathogenic (variant classification). For the other abbreviations see Table 1.

**Table 4:** Current and emerging genotype-directed therapeutic approaches.

Gene/Target	Therapy	Mechanism
Sarcomeric (MYH7, MYBPC3, etc.) [108]	Mavacamten	Myosin ATPase inhibitor reduces hypercontractility.
Sarcomeric/HFrEF (mixed) [109]	Omecamtiv mecarbil	Myosin activator increases systolic function
LMNA [110,111]	ARRY-371797 (PF-0726580)	p38 MAPK inhibition reduces stress/fibrosis.
LMNA [112]	PHL-001 (shRNA)	Allele-specific LMNA silencing.
FLNC truncating variants [113]	Crenolanib	PDGFRA inhibition improves FLNCtv. iPSC-CM function/arrhythmias
BAG3 [112]	AVB-401	BAG3 gene-replacement vector.
PKP2 [112]	TN-401	PKP2 gene-replacement vector.
MYH7 [114,115]	Gene editing/replacement	Corrects MYH7 sarcomeric mutations.
RBM20 [116]	Gene therapy/editing	Restores normal splicing (TTN and others).

HFrEF: heart failure with reduced ejection fraction; iPSC-CM: induced pluripotent stem cell-derived cardiomyocyte. PDGFRA: Platelet-Derived Growth Factor Receptor Alpha; PHL-001: short hairpin RNA investigational agent. For the other abbreviations see Table 1.

## Data Availability

No new data were created or analyzed in this study. Data sharing is not applicable to this article.

## Abbreviations

The following abbreviations are used in this manuscript:

AAV	adeno-associated virus
ACM	Arrhythmogenic cardiomyopathy
ACMG/AMP	American College of Medical Genetics and Genomics and the Association for Molecular Pathology
ARVC	arrhythmogenic right ventricular cardiomyopathy
BAG3	Gene encoding for Bcl2-associated athanogene 3
DCM	dilated cardiomyopathy
DES	Gene encoding for desmin
DSP	Gene encoding for desmoplakin
DSC2	Gene encoding for desmocollin 2
DSG2	Gene encoding for desmoglein 2
FLNC	Gene encoding for filamin C
HCM	hypertrophic cardiomyopathy
HF	Heart failure;
ICD	implantable cardioverter–defibrillator
iPSC-CM(s)	induced pluripotent stem cell-derived cardiomyocyte(s)
LVEF	left ventricular ejection fraction
LMNA	Gene encoding for lamin A/C
LOS	Losso of function
MYH7	Gene encoding for myosin heavy chain 7
NDLVC	non dilated left ventricular cardiomyopathy
NGS	next-generation sequencing
LOF	predicted loss-of-function
PDGFRA	platelet-derived growth factor receptor-α
PHL-001	short hairpin RNA investigational agent
PKP2	Gene encoding for pakophilline 2
PLN	Gene encoding for phospholamban
PRSs	polygenic risk scores
P/LP	pathogenic/likely pathogenic (variant classification)
PPCM	Peripartum cardiomyopathy
RBM20	Gene encoding for RNA binding protein 20
RCM	Restrictive cardiomyopathy
SCD	sudden cardiac death
SCN5A	Gene encoding for Sodium channel protein type 5 subunit α
SERCA	sarcoplasmic reticulum Ca2+-ATPase
TNNT	Gene encoding for troponin
TPM	Gene encoding for tropomiosin
TTN	Gene encoding for titin
WES	whole-exome sequencing
